# Grayken lessons: the role of an interdisciplinary endocarditis working group in evaluating and optimizing care for a woman with opioid use disorder requiring a second tricuspid valve replacement

**DOI:** 10.1186/s13722-023-00360-7

**Published:** 2023-02-07

**Authors:** Hallie Rozansky, Eric Awtry, Zoe M. Weinstein, Alyssa F. Peterkin

**Affiliations:** 1 Grayken Center for Addiction, Clinical Addiction Research and Education Unit, Section of General Internal Medicine, Department of Medicine, Boston Medical Center, and the Boston University Chobanian & Avedisian School of Medicine, 801 Massachusetts Ave. Crosstown Building, 2nd Floor, Boston, MA 02118 USA; 2 Section of Cardiology, Department of Medicine, Boston Medical Center, and the Boston University Chobanian & Avedisian School of Medicine, Boston, MA 02118 USA

**Keywords:** Infective endocarditis, Substance use disorder, Harm reduction, Endocarditis team, Endocarditis working group

## Abstract

**Background:**

Injection drug use-related endocarditis is increasingly common among hospitalized patients in the United States, and associated morbidity and mortality are rising.

**Case presentation:**

Here we present the case of a 34-year-old woman with severe opioid use disorder and multiple episodes of infective endocarditis requiring prosthetic tricuspid valve replacement, who developed worsening dyspnea on exertion. Her echocardiogram demonstrated severe tricuspid regurgitation with a flail prosthetic valve leaflet, without concurrent endocarditis, necessitating a repeat valve replacement. Her care was overseen by our institution’s Endocarditis Working Group, a multidisciplinary team that includes providers from addiction medicine, cardiology, infectious disease, cardiothoracic surgery, and neurocritical care. The team worked together to evaluate her, develop a treatment plan for her substance use disorder in tandem with her other medical conditions, and advocate for her candidacy for valve replacement.

**Conclusions:**

Multidisciplinary endocarditis teams such as these are important emerging innovations, which have demonstrated improvements in outcomes for patients with infective endocarditis and substance use disorders, and have the potential to reduce bias by promoting standard-of-care treatment.

## Background

Injection drug use (IDU)-related endocarditis is increasingly common in the United States, and innovative care delivery methods are needed to provide equitable treatment to some of our highest-risk patients with substance use disorders (SUDs). Here we present the case of one such patient who experienced complications of IDU-related endocarditis. We follow this with expert commentary from two specialists at our institution, regarding their experiences developing and working with our multidisciplinary Endocarditis Working Group. This team was created in 2017 in order to standardize and optimize care for patients with IDU-related endocarditis, in response to provider and patient perceptions that some patients were not receiving medically necessary valve surgery, and concerns that treatment decisions for patients with SUDs were driven by stigma rather than by guidelines. Finally, we conclude with lessons learned and implications for other institutions seeking to improve care for patients with IDU-related endocarditis.

## Case information

Ms. D, a 34-year-old woman with severe opioid use disorder (OUD), was admitted to an academic medical center for elective replacement of her prosthetic tricuspid valve. Three years prior, she had undergone prosthetic valve replacement for methicillin-susceptible staphylococcus aureus endocarditis, which required urgent intervention due to the presence of large vegetations and severe tricuspid regurgitation.

After her first episode of endocarditis, she continued to inject fentanyl. She developed three additional episodes of IDU-associated prosthetic tricuspid valve bacterial endocarditis, as well as several episodes of culture-negative endocarditis, for which she received antibiotics. She tried methadone and buprenorphine-naloxone as medications for opioid use disorder (MOUD), and they did not initially reduce her non-prescribed opioid use. About 2 years later, in the setting of increased attendance at her methadone maintenance program, she stopped using injection opioids.

Around the same time, she began developing progressive dyspnea. She had an outpatient echocardiogram, which revealed recurrent severe tricuspid regurgitation with a flail prosthetic valve leaflet and worsening right ventricular systolic function, without evidence of active endocarditis. She thus underwent evaluation for a prosthetic valve replacement.

Ms. D’s past medical history was otherwise notable for chronic hepatitis C and septic pulmonary embolism, secondary to IDU, as well as left heart failure with preserved ejection fraction, thought to be attributable to IDU as well as obesity. Mental health conditions included depression, anxiety, and post-traumatic stress disorder. She lived with her husband in an apartment. Her family history included alcohol and cocaine use disorders, as well as unspecified mental health disorders in her mother and sister.

Her substance use history included intranasal and injection use of fentanyl and cocaine, with a lifetime history of two opioid overdoses, once requiring naloxone. She smoked several cigarettes daily and occasionally used cannabis. When she first started MOUD with methadone, she frequently missed doses. However, at the time of her evaluation for prosthetic valve replacement, she had been abstinent from non-prescribed opioids for 1 year. She was in good standing at her methadone maintenance program, where she was prescribed 77 mg methadone daily with 13 “take-homes.” Take-homes, doses of methadone that can be self-administered in an unobserved setting, are provided to clients demonstrating moderate adherence to methadone and stability in their recovery, and reduce the frequency with which clients must present to the clinic. Notably, Ms. D’s take-homes were issued in the setting of the Substance Abuse and Mental Health Services (SAMHSA) exception expansion policies implemented during the COVID-19 pandemic. This expansion allowed flexibility in prescribing take-homes to patients who were less stable in their recovery but were medically complex and at high risk for complications of COVID-19. Early evidence suggests that these expanded take-homes increase treatment engagement and patient satisfaction, with minimal negative consequences [1, 2].

Boston Medical Center’s Endocarditis Team, known as the “Endocarditis Working Group,” oversaw Ms. D’s evaluation for valve replacement. This team was developed in 2017 in order to promote evidence-based treatment for patients with IE and minimize the extent to which stigma influenced care for PWID, and includes multidisciplinary providers from cardiology, cardiothoracic surgery, addiction medicine, infectious disease, and neurocritical care. Ms. D was initially connected to the working group through her outpatient cardiologist. As part of her evaluation by the Working Group, Ms. D attended outpatient appointments with infectious disease, cardiothoracic surgery, and addiction medicine. She also saw her primary care provider, who was aware of the ongoing evaluation and was supportive.

Based on Ms. D’s symptoms and echocardiographic findings, a valve replacement was recommended. Because she did not meet criteria for an urgent valve replacement (e.g., overt heart failure, heart block, ongoing infection despite appropriate antibiotic therapy, large mobile vegetation, recurrent embolic phenomena) [3], but rather had compensated sequelae of prior IE, the Working Group was able to follow her longitudinally. They collaborated with her methadone maintenance program and assessed her stability in recovery, which included consideration of her “recovery capital” (family support, stable housing, time in recovery, among others) as well as her adherence to MOUD. After sequential evaluations, the Working Group team members determined that the benefits outweighed the risks of a procedural intervention. She thus underwent a transcatheter tricuspid valve-in-valve replacement, chosen instead of an open surgical procedure due to the risks of complications associated with second sternotomy.

Ms. D was admitted to the hospital post-procedurally for monitoring, and at that time, her husband disclosed that she had stopped taking methadone 1 week prior, unbeknownst to her providers. The primary team called the addiction consult team for assistance, to whom Ms. D affirmed her wish to maintain opioid abstinence without the use of MOUD. Before an alternate treatment plan was arranged, she left the hospital via patient-directed discharge.

The addiction provider from the Endocarditis Working Group, who had been in touch with the addiction consult team during the admission, reached out to Ms. D and scheduled close outpatient follow up. At her outpatient visit, Ms. D started oral naltrexone, per her preference. Shortly after, due to persistent opioid cravings, she began purchasing non-prescribed methadone while awaiting re-admission to a methadone maintenance program.

The addiction provider collaborated with a local methadone maintenance program to expedite Ms. D’s admission to the clinic, and communicated frequently with Ms. D throughout this time period. Ms. D’s outpatient cardiology and psychiatry providers also followed closely, rescheduled missed appointments as needed, and provided transportation assistance. At that time, the Working Group did not have a patient navigator, thus care coordination fell to individual outpatient providers and their clinical staff. Ms. D unfortunately did not qualify for additional home support, such as a visiting nurse.

While awaiting admission to the methadone clinic, Ms. D used intranasal fentanyl, which led to an overdose requiring naloxone reversal. After her non-fatal overdose, she successfully re-engaged in her methadone maintenance program. She has not had any additional episodes of endocarditis or worsening heart failure and remains on MOUD.

## Expert commentary

The following commentary is provided by two physicians:

1. Dr. Eric Awtry, MD, Associate Professor of Medicine at the Boston University Chobanian & Avedisian School of Medicine and Associate Chief for Clinical Affairs for the Section of Cardiology at Boston Medical Center. Dr Awtry serves as the cardiology lead on the Endocarditis Working Group.
2. Dr. Zoe Weinstein, MD, MS, Assistant Professor of Medicine at the Boston University Chobanian & Avedisian School of Medicine, Director of the Addiction Consult Service, and Associate Program Director of the Addiction Medicine Fellowship at Boston Medical Center. Dr. Weinstein serves as the addiction medicine lead on the Endocarditis Working Group.

*To Dr. Awtry: Can you tell us a little more about why the Endocarditis Working Group, was founded, and how it is structured?*

Our group was founded in November 2017 in response to our perception that treatment of patients with IE, especially for PWID, was not always optimal. Decisions regarding candidacy for valve replacement in PWID were somewhat subjective, non-uniform, occasionally unilateral, and not always timely. In addition, there were often conflicting recommendations from different services. This resulted in missed opportunities for care and very few PWID underwent corrective surgery. We formed the Working Group to bring experienced clinicians together to evaluate complex patients and facilitate optimal care. Our goals included standardizing decision-making for patients with IE, identifying barriers to optimal care and developing solutions, monitoring progress and outcomes for patients with IE, and providing a forum where other services could present patients and receive a rapid consensus decision regarding optimal treatment plans. Notably, there are multiple different published models for endocarditis teams, and ours represents only one particular structure [4].

From the onset, we envisioned a multidisciplinary approach to the Working Group, drawing on expertise from all specialties essential to the care of our patients with IE. The core membership in the group has always included physicians from cardiology, cardiac surgery, infectious disease, addiction medicine, and neurology. In addition, we have drawn on our colleagues from pharmacy, internal medicine, and medical subspecialties to assist in developing systems to support patient care or in evaluating specific patients. The team meets for one to one-and-a-half hours biweekly, and if an urgent need arises between scheduled meetings, the team can assemble ad hoc. The team members do not necessarily see and care for the patients themselves, but collaborate closely with and convey recommendations to the patients’ primary providers.

Patients who do not require urgent intervention are often longitudinally evaluated in the outpatient setting by multiple subspecialists. Given the psychosocial challenges that affect many patients with IE, we support patients by obtaining transportation assistance if needed, using telemedicine visits when possible and appropriate, and being flexible with rescheduling missed appointments. Until recently, the individual providers in the Working Group and their clinic staff were tasked with the challenge of ensuring that patients came to their appointments and contacting them when they were lost to follow up. As of 2022, we have added a patient navigator to the Working Group, who can now coordinate across disciplines to provide this support.

*To Dr. Weinstein: How has care for PWID with infective endocarditis changed, from prior to establishment of the Endocarditis Working Group to now?*

We have not yet undertaken a formal evaluation of patient outcomes since establishment of this Working Group, but in my subjective experience, as both an addiction specialist and a primary care provider, this has been transformative.

For many years, both patients and providers felt that stigma was guiding care, and that PWID with IE were often not offered surgery unless they were extremely ill, and perhaps not even then. Now, we focus first and foremost on whether the patient has an indication for surgery, and whether this indication is urgent or elective. If we agree that surgery is indicated, then we work together to optimize the patient to make that procedure as successful as possible in the short- and long-term. We have helped our colleagues appreciate that SUDs are chronic, relapsing, and treatable diseases, and we are a valued part of the group. We and our patients now feel that all their providers are their allies and are on the same team—the patient’s team.

On the rare, sad occasions when I am told that my patient is not a surgical candidate, I now know that the patient has been fully and objectively evaluated, and that this decision represents the fact that there is true medical futility in the procedure (anatomically impossible, too risky due to recent strokes, etc.). This allows me to confidently transition to conversations about other next steps, including palliative care.

*To Dr. Awtry: How do you and your colleagues on the Endocarditis Working Group evaluate whether a PWID is a candidate for valve surgery? What about when considering someone for a second, or third valve?*

The first step is identifying patients with IE. In both the outpatient and inpatient settings, providers who care for patients with IE refer them ad hoc to our Working Group, with most referrals from cardiology and infectious disease. More recently, we have also developed order sets in the electronic medical record for suspected and confirmed endocarditis, to streamline initial patient management and encourage early involvement of the specialties represented in the Working Group.

Once referred to our group, patients are presented at our biweekly meetings by the patient’s primary team or a member of the Working Group. Each Working Group member weighs in. Infectious disease comments on the likelihood of success with conservative management, based on the specific organism involved and any evidence of antimicrobial failure. Cardiology reviews echocardiographic findings and discusses structural or functional indications for surgery. Cardiac surgery discusses the technical feasibility of surgery and the impact of comorbidities on post-operative recovery. Addiction medicine discusses the patient’s SUD, recovery capital, and likelihood of post-operative abstinence. Neurology discusses the need for further imaging and the ideal timing of surgery for patients with central nervous system embolic events. Outpatient primary care providers are not formally involved in the Working Group meetings, but we often contact them if we have questions or to collaborate.

We strive to apply evidence-based and/or consensus guidelines to the care of our patients, and have developed algorithms to facilitate our discussions. Different considerations apply for acute versus subacute, IDU-associated versus non-IDU-associated, and right-sided versus left-sided IE. The primary consideration, however, is whether the patient has an urgent or elective indication for surgery.

Once the group reaches a consensus, we make recommendations to optimize the patient pre-operatively, or outline the appropriate therapeutic plan and clinical follow-up for patients for whom conservative therapy is indicated. If the patient is hospitalized, we convey our recommendations to the primary team and relevant consultants. If the patient is an outpatient, we reach out to their primary care physician, and the subspecialists from the Working Group typically become part of their longitudinal outpatient team of providers. In the event that a patient requires surgical valve repair or replacement, a cardiac surgeon affiliated with the Working Group will personally perform the procedure. For transcatheter treatments (transcatheter valve replacement or vacuum vegetectomy), the Working Group members may not perform the procedure themselves, but will communicate directly with the interventional cardiologists to advocate and share the Group’s recommendations.

For repeat valve replacements, our approach is similar to that of our primary evaluation, and also takes into account the cause of the recurrent endocarditis. There is substantially increased risk associated with a second or third surgery, and at times, non-operative therapy may offer lower risk, even in patients with clear surgical indications. The majority of repeat infections are related to recurrent IDU and in those circumstances, input from addiction medicine is essential. Often, psychosocial stressors are present and place the patient at risk of future IDU and associated infections. Other times, addiction treatment may not have been provided or optimized at the time of initial valve surgery. These and other considerations weigh into the decision to offer repeat valve surgery.

*To Dr. Weinstein: How do you optimize a patient’s recovery in their substance use disorder in preparation for valvular surgery?*

We try to be very patient-centered, and of course utilize evidence-based treatments. First, we make sure that the patient understands the relationship between substance use and IE, the risk of recurrent IE with ongoing use, and the potential for more limited surgical options in the future.

In treating SUDs, we try to use all the tools available. This starts with harm reduction. We discuss harm reduction principles, understanding that addiction is a chronic, relapsing disease, emphasizing the need to use sterile injection equipment if the patient returns to IDU, and reviewing other ways to decrease risk of infection (e.g., using intranasally instead of intravenously) to prevent recurrent IE.

Most PWID with IE in our patient population have OUD, though some patients have other primary or co-occurring SUDs. We thus focus heavily on evidence-based MOUD—specifically, the opioid agonists, methadone and buprenorphine. For patients not on MOUD, we discuss starting these medications. If patients are already on MOUD, we work on optimizing their medication, ensuring that they are on therapeutic doses, and navigating challenges related to stabilization of their regimen. If the patient has other active SUDs, we ensure they are offered other medications, as appropriate. We also offer psychosocial support, working to link patients to outpatient counseling or residential treatment if these supports will help sustain them in recovery.

The Working Group’s addiction providers can directly manage our patients’ addiction care or can support their local care providers in doing so if that is preferred. Some patients live far from the hospital, and so it is not practical for them to receive routine addiction care in our system. Additionally, our hospital does not have its own methadone maintenance program, so all patients on methadone receive care outside of our hospital system. In these cases, members of the Working Group, like myself, can see the patient in our hospital-based outpatient clinic for peri-operative consultations. We perform an addiction-focused history and physical exam, and obtain consents to reach out to the patient’s local addiction treatment providers. We then review records or verbally confirm treatment history, and advocate for optimizing addiction treatment, if appropriate. For example, we may recommend medication dose adjustments or increased counseling services.

*To Dr. Weinstein: Is MOUD considered a prerequisite for valvular surgery in patients with OUD in the Endocarditis Working Group?*

We aim to be patient-centered and evaluate each patient individually, so we do not have any absolute criteria. Our goal is to optimize patients for short- and long-term health and recovery. For many patients, that includes MOUD. For patients in recovery without MOUD, we discuss that upcoming post-operative pain and pain medications can be potential triggers for returning to use, and ask whether, in that setting, they think they would benefit from initiating MOUD in the peri-operative period. However, we do not require that patients be on MOUD to receive surgery.

*To Dr. Awtry: What do you see as goals for future improvement for the working group? If other institutions were interested in starting similar groups, what words of advice might you share?*

Our group is comprised of volunteer physicians. Time is often a challenge, and biweekly meetings are not always sufficient. In addition, while the Working Group is a resource to assist in the care of patients with IE, its members are not necessarily part of the patient’s inpatient or outpatient care teams. Other institutions have services that formally round and consult on all inpatients with IE; we could consider a similar model, but it would require significantly more physicians and hospital support.

We have difficulty keeping close contact with some of our patients and ensuring that they receive ongoing care. As of 2022, we received a grant to fund a patient navigator, who has taken on much of this responsibility. This navigator reaches out to patients in the hospital and in the community, helps to schedule and reschedule peri-operative visits, secures support services for those in need, facilitates ongoing communication between the patients and the care team and, in doing so, fosters the development of a trusting relationship that is essential for the care of these patients.

Finally, we have not yet formally evaluated the Working Group. However, we are currently developing a hospital-supported retrospective and prospective database of patients with IE, which will allow for assessment of outcomes and quality improvement projects, and provide the foundation for clinical research as we continue to strive to improve patient care.

For institutions interested in starting a similar group, I would offer the following pieces of advice:

1. The team needs to be multidisciplinary and include representatives from the specialties noted previously. The members should not only be knowledgeable in their fields but also interested in committing their time and willing to work collaboratively.
2. The working group should not only review cases and generate consensus recommendations, but should also identify gaps in the care of their patients and develop systems for improvement.
3. For these teams to be most effective, institutions need to offer support, providing the time for physicians to work on these teams and the administrative assistance to help very complicated patients navigate a very complicated system.

## Lessons learned

1. Infective endocarditis is increasing in prevalence among PWID and is associated with significant morbidity and mortality.

IE is approximately 100 times more common in PWID than in the general population and rates have been rising. From 2007 to 2017, in North Carolina, PWID went from comprising 6% to 42% of IE surgeries in the state [5]. Over the last two decades, the demographics of PWID with IE have also shifted, to include greater proportions of patients who are younger, white, and female [6, 7]. The reasons behind gender-associated differences are unknown, but may be explained by women representing an increased proportion of PWID overall, as well as evidence that women may be more likely to borrow and share injection equipment, and differences in injection technique between genders [7, 8]. In contrast, IE in people who do not use drugs tends to affect more older patients, more men, and more Black and Hispanic patients [5].

Mortality of IE in PWID is also rising. From 1999 to 2016, there was a three-fold increase in deaths due to IE in PWID, compared to a one-and-a-half-fold increase in the general population. This mortality increase was particularly evident in those younger than 35 years old, for whom mortality increased from 12.4 to 37.4% [9]. Modeling predicts that more than 250,000 PWID will die from IE from 2020 to 2030 [10].

PWID who require surgery for endocarditis have lower operative and early post-operative morbidity and mortality than patients with IE who do not inject drugs, likely reflecting a younger population with fewer comorbidities [11–14]. However, PWID experience more delayed post-operative complications, with higher rates of reoperation, valvular complications, and recurrent endocarditis [12, 14–18]. This is at least partly due to ongoing drug use [17, 19].

2. Strategies that show promise in reducing morbidity and mortality of IE in PWID, including MOUD, referral to addiction treatment, and safe injection techniques, are underutilized.
  *Medications for opioid use disorder*
  MOUD are effective in reducing non-prescribed opioid use, improving retention in addiction treatment, and decreasing overall mortality in PWID [20]. Multiple studies have examined the impact of MOUD on mortality specifically among PWID with IE; these studies have either shown a non-significant trend toward benefit or no difference, which may be due to underpowering and substantial study limitations, such as using very low doses of methadone or using buprenorphine only in patients not concomitantly receiving opioids for pain [21–23]. Very few PWID with IE even receive MOUD, ranging from less than 12% to 25%, further limiting available data on outcomes [14, 22].
  *Referral to addiction treatment*
  Referral to comprehensive addiction treatment has been shown to decrease 1-year mortality for PWID with IE by up to 70% and to be protective against 2-year mortality in post-operative patients [24, 25]. Inpatient addiction treatment is associated with a lower incidence of new bloodstream infections in this population [26]. Furthermore, PWID with serious infections who receive inpatient addiction medicine consultation are significantly more likely to receive MOUD, finish their antibiotics, and remain hospitalized [27]. However, delivery of addiction care to PWID with IE is inconsistent, with fewer than 25% of patients receiving addiction referrals or consultations [24, 25, 28]. In a survey of cardiac surgeons, only 35% reported having addiction services at their hospitals, but 93% reported that they engaged addiction services for patients with IDU-associated IE when available [29].
  *Safer injection practices*
  Finally, the use of safer injection practices could dramatically reduce death from IE. A 2021 simulation estimated that men who frequently used opioids via “high-risk injection techniques” (defined as high-frequency use with sharing of non-sterile equipment) had a 44–54% probability of dying from IE by age 60. However, using sterile injection technique and avoiding needle sharing reduced this probability to 2–4% [10]. Reducing frequency of injection use, without cessation of use, has been shown to reduce the risk of bacterial infections by 36% [30]. One study demonstrated that PWID who used sterile injection equipment were significantly less likely to have IE [7].

3. Multidisciplinary Endocarditis Working Groups are emerging interventions that can improve care for PWID with IE.

The American Association for Thoracic Surgery (AATS) has consensus guidelines on the indications for surgical treatment of endocarditis [3]. However, stigma and discrimination toward PWID, as well as concerns about long-term clinical outcomes, have fueled debate as to whether PWID should be considered for endocarditis surgery [31–34]. Multiple clinical guidelines suggest denying PWID surgery, even for patients meeting AATS criteria [35]. Consequently, providers for PWID have sought opportunities to advocate for equitable evaluation and access to surgery.

One such emerging strategy is the development of institutional Endocarditis Working Groups. These teams are multidisciplinary groups that provide early detection and management of IE, establish guideline-based standardized protocols for evaluation of surgical candidacy, and optimize treatment for SUDs to help patients achieve stability in recovery and maximally benefit from medical or surgical interventions. Endocarditis teams ideally have the ability to provide longitudinal care and provide wraparound inpatient and outpatient support. They have demonstrated improvements in outcomes, including earlier addiction medicine consultation and increased MOUD prescriptions, as well as reductions in in-hospital mortality, 3-year mortality, surgical mortality, length of stay, time to antibiotics, time to surgery, and embolic events, among other outcomes [36–40]. Drawing on this evidence, the American Heart Association released a statement in 2022 declaring that “multispecialty endocarditis teams are pivotal in the care of patients with IE,” and declared addiction medicine expertise “critical” when caring for patients with IDU-associated IE [41].

## Conclusion

Over the past two decades, the incidence and mortality of IE have surged in PWID, especially in young, female patients. PWID often experience barriers when seeking surgical treatment for IE due to higher rates of delayed post-operative complications and stigma, and often receive suboptimal care, including low rates of linkage to addiction treatment programs and prescription of MOUD. Endocarditis Working Groups are innovative teams that bring together multidisciplinary providers to evaluate and optimize the care of complex patients with IE and have demonstrated improvements in outcomes. For patients such as Ms. D, the Endocarditis Working Group was fundamental in systematically evaluating her case, connecting her to key providers, establishing her eligibility for valve replacement, and then helping her reconnect to care, restart MOUD, and re-achieve stability after she was transiently lost to follow-up.

## Data Availability

Not applicable.

## Abbreviations

IE: Infective endocarditis
IDU: Injection drug use
MOUD: Medications for opioid use disorder
OUD: Opioid use disorder
PWID: Person or people who inject(s) drugs
SUD: Substance use disorder
